# Healthcare leaders’ experiences of implementing artificial intelligence for medical history-taking and triage in Swedish primary care: an interview study

**DOI:** 10.1186/s12875-024-02516-z

**Published:** 2024-07-24

**Authors:** Elin Siira, Daniel Tyskbo, Jens Nygren

**Affiliations:** School of Health and Welfare, Halmstad University, Box 823, Halmstad, 301 18 Sweden

**Keywords:** Implementation, Artificial intelligence, Medical history-taking, Triage, Primary care, Healthcare leaders

## Abstract

**Background:**

Artificial intelligence (AI) holds significant promise for enhancing the efficiency and safety of medical history-taking and triage within primary care. However, there remains a dearth of knowledge concerning the practical implementation of AI systems for these purposes, particularly in the context of healthcare leadership. This study explores the experiences of healthcare leaders regarding the barriers to implementing an AI application for automating medical history-taking and triage in Swedish primary care, as well as the actions they took to overcome these barriers. Furthermore, the study seeks to provide insights that can inform the development of AI implementation strategies for healthcare.

**Methods:**

We adopted an inductive qualitative approach, conducting semi-structured interviews with 13 healthcare leaders representing seven primary care units across three regions in Sweden. The collected data were subsequently analysed utilizing thematic analysis. Our study adhered to the Consolidated Criteria for Reporting Qualitative Research to ensure transparent and comprehensive reporting.

**Results:**

The study identified implementation barriers encountered by healthcare leaders across three domains: (1) healthcare professionals, (2) organization, and (3) technology. The first domain involved professional scepticism and resistance, the second involved adapting traditional units for digital care, and the third inadequacies in AI application functionality and system integration. To navigate around these barriers, the leaders took steps to (1) address inexperience and fear and reduce professional scepticism, (2) align implementation with digital maturity and guide patients towards digital care, and (3) refine and improve the AI application and adapt to the current state of AI application development.

**Conclusion:**

The study provides valuable empirical insights into the implementation of AI for automating medical history-taking and triage in primary care as experienced by healthcare leaders. It identifies the barriers to this implementation and how healthcare leaders aligned their actions to overcome them. While progress was evident in overcoming professional-related and organizational-related barriers, unresolved technical complexities highlight the importance of AI implementation strategies that consider how leaders handle AI implementation in situ based on practical wisdom and tacit understanding. This underscores the necessity of a holistic approach for the successful implementation of AI in healthcare.

**Supplementary Information:**

The online version contains supplementary material available at 10.1186/s12875-024-02516-z.

## Background

Efficient medical history-taking and triage are crucial in primary care to optimise resource allocation, meet patient needs, and ensure the safe and effective delivery of healthcare. The process involves a primary care professional assessing a patient’s history either over the phone or in-person to determine the best course of action [1–3]. However, the increasing demand for medical consultations globally, coupled with a decline in the primary care workforce, has created a challenging work environment for primary care providers [4, 5]. This strain may result in suboptimal over-the-phone medical history-taking, potentially endangering patient safety [2, 6–9].

Artificial intelligence (AI) has been proposed as a potential solution to automate medical history-taking and triage, which could reduce the workload of primary care professionals and improve safety and efficiency [10–14]. Unlike many commonly studied digital technologies, AI’s primary role is not merely to structure and convey data and information but to interpret it and actively contribute to the creation and evaluation of new knowledge claims [15, 16]. This unique function serves to support healthcare professionals in their core medical responsibilities and in their efforts to optimize care, rather than solely functioning as a tool for administrative tasks and for improving efficiency [17, 18].

Previous research has identified a range of barriers to the implementation of AI in healthcare [19, 20] and more specifically AI applications for medical history-taking and triage [13, 14, 21, 22]. These encompass a spectrum of challenges, including concerns about the performance of AI systems [13, 14, 21], compatibility issues with existing technical infrastructures [22], healthcare professionals’ hesitancy to embrace AI-generated diagnoses [21], negative attitudes among healthcare practitioners [22], and time constraints impeding the use of AI systems [21]. The barriers to implementing AI are notably more complex when compared to other digital technologies, stemming from the intricacy, unpredictability, limited empirical evidence, and the challenge in comprehending what AI is [23].

The quest to understand how to effectively surmount implementation barriers remains an underexplored aspect within the realm of AI implementation in healthcare [24–26]. Empirical investigations into the adoption of innovations, particularly digital triage systems deployed in primary care, have consistently highlighted the pivotal role played by healthcare leaders in the deployment and success of such systems [27–31]. For instance, in the realm of digital technology innovations, leaders play a crucial role in framing and managing meaning to enhance motivation and foster collective action [32]. In primary care, leaders are also often the ones making decisions to implement or disregard new solutions [28, 30, 33, 34]. Moreover, existing implementation frameworks consistently emphasize the critical impact of healthcare leaders and their facilitative actions in shaping implementation outcomes [35–37] through their role in diffusing information, synthesizing strategies, and mediating between organisational goals and daily operations [38]. Reviews of contextual factors influencing implementation outcomes have highlighted that while sufficient financial resources and time availability are favourable conditions for implementation, their effectiveness is greatly enhanced when combined with supportive leadership and positive social relations [35, 36]. This suggests that leadership not only plays a significant role in allocating resources but also in fostering a conducive environment for successful implementation efforts. Based on these insights, it is plausible to assume that healthcare leaders play a crucial role in the successful implementation of AI applications for medical history-taking in triage in primary care.

However, there is a gap in the current body of research regarding healthcare leaders’ experiences of implementing AI in the primary care context [3], as well as in other healthcare settings [39, 40]. This knowledge gap is further compounded by a paucity of insights into how healthcare leaders effectively address the barriers tied to AI implementation and the approaches they employ to overcome them [18]. To gain insights into what works in specific settings, it is essential to examine and acknowledge how healthcare leaders address the unique implementation challenges of their contexts [41].

In this paper, we address this critical gap in knowledge by exploring healthcare leaders’ experiences of implementing an AI application for automating medical history-taking and triage within primary care settings, the barriers they faced and the actions they took to navigate around these barriers. In line with a growing trend in leadership research, this paper adopts a practice-based definition of leaders. It focuses on individuals actively engaged in leadership activities, solely than only relying on formal titles or roles [42, 43]. This inquiry is paramount because, despite the pledged advancements of AI in healthcare, its actual implementation remains uncharted territory, hindering the realization of its potential to improve healthcare delivery [39, 42, 43].

Our research aims to provide valuable insights into the actions taken by healthcare leaders that serve as the foundation of organizational advancement [44]. By doing so, we seek to provide important knowledge that can inform the creation of AI implementation strategies, ultimately advancing the field of AI in primary care towards greater efficiency and success. The implementation of AI has to date often lacked explicit guidance for healthcare leaders during the implementation process [18, 39, 40, 45]. Healthcare leaders often rely on their own experiences and actions to gradually develop intentional strategies when organizations lack clearly defined strategies. Empirical research can uncover this type of activities that prompt the creation of organizational strategies [44, 46–48]. Knowledge about these activities can serve as building blocks for successful implementation of AI applications in healthcare, including for medical history-taking and triage in primary care.

This study explores the first-hand experiences of healthcare leaders in Sweden who undertook the implementation of an AI application for automating medical history-taking and triage in primary care. We analyse the encountered barriers and the actions they took to navigate around these impediments. This exploration is guided by the following two research questions:

1. Which barriers were confronted by healthcare leaders in the process of implementing the AI application for automating medical history-taking and triage in primary care?
2. Which actions were employed to effectively navigate around and overcome these encountered barriers?

## Method

### Design

We utilized an inductive qualitative approach in this interview study. We conducted individual semi-structured interviews [49] to retrospectively gather healthcare leaders’ experiences of the implementation of an AI application for medical history-taking and triage. Thematic analysis [50], which has commonly been employed in research on stakeholders’ experiences of implementing new technology in healthcare (see for instance, [51–53]), was used to analyse the interview data. The study is reported in accordance with the Consolidated Criteria for Reporting Qualitative Research (COREQ) [54] to ensure clear and comprehensive reporting (see Additional file 1).

### Setting

#### The Swedish context

The Swedish government has actively fostered data-driven and digital-driven innovations in public services through a range of initiatives [55]. Publicly funded healthcare is administered at state, regional, and municipal levels in Sweden. The nation’s 21 regions predominantly oversee healthcare delivery, focusing on health promotion, disease prevention, and treatment of injuries and illnesses. Notably, the introduction of regional digital primary care in 2016 marked a significant expansion [56, 57], with AI utilization concentrated in domains such as medical history-taking, diagnosis, and decision support [58].

#### The AI application for medical history-taking and triage

**Fig. 1:**
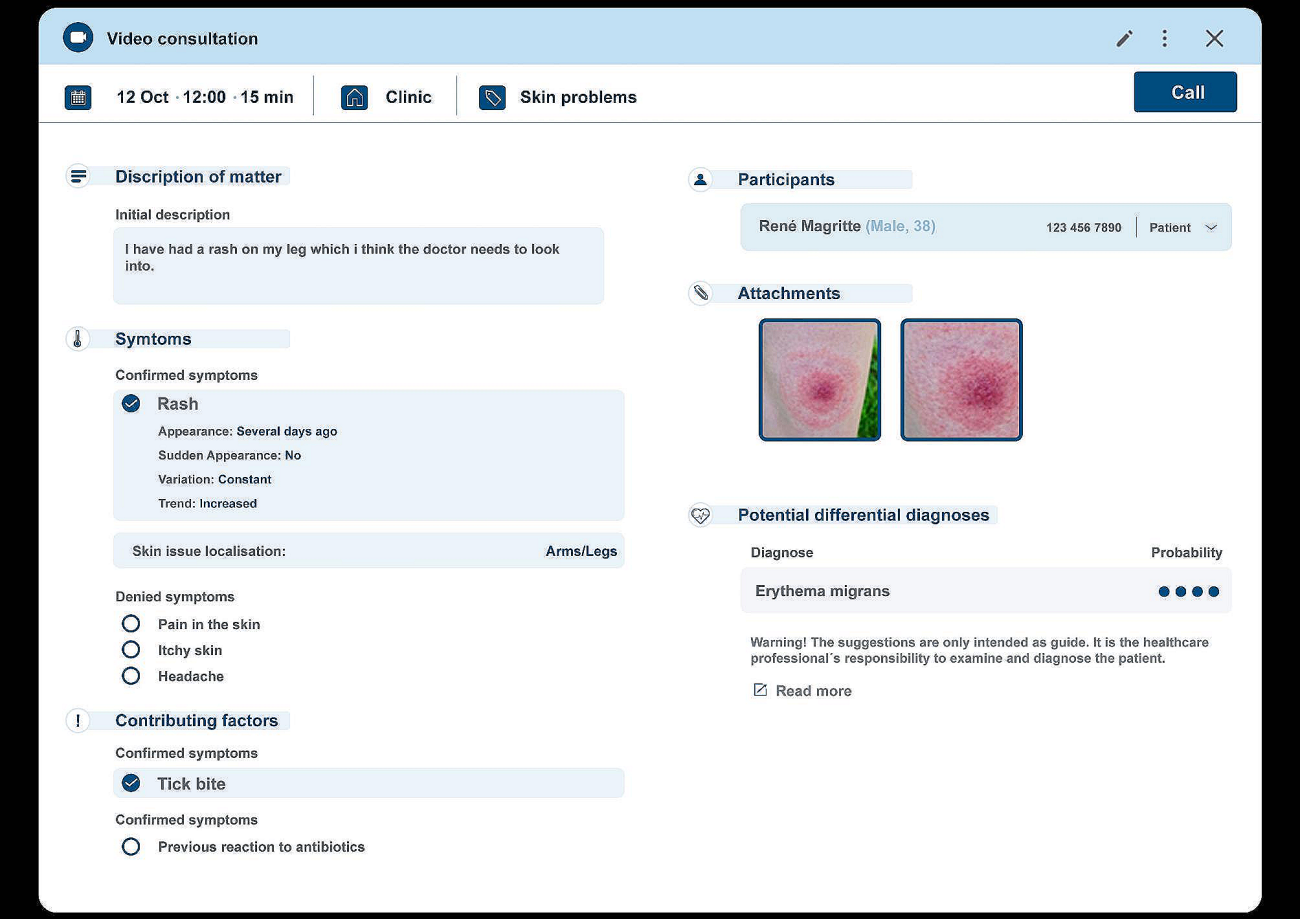
Summary of the information provided by the AI application

The AI system that is fundamental for this study is a software application designed for the automation of medical history-taking and triage and based on a Bayesian network framework. At the time of the study, its implementation and utilization spanned public primary care facilities across four regions in Sweden. The primary objective of this AI system is to automate the triage process while providing support for clinical decision-making and patient management within the context of primary care. Its underlying aim is to augment the reliability and efficiency of these processes. When patients access primary care services online, the AI application collects their medical history and triage information through an automated chat interface. The AI then generates a comprehensive report detailing the patient’s symptoms, the urgency of their condition, and a list of potential differential diagnoses. This report serves as a resource for primary care professionals, who make the ultimate decisions regarding the patient’s care plan. For an illustrative representation of the AI application’s output as presented to primary care professionals, please refer to Fig. 1.

### Participants

At the time of the data collection, the AI application was implemented or used in four Swedish healthcare regions. Participants were recruited from three of these healthcare regions, reflecting a purposive sampling approach. The included regions represent a diverse range of characteristics, including both small and large regional organizations, urban and rural geographical locations, and small and large populations. Eligibility criteria for inclusion encompassed individuals who fulfilled the following conditions: (1) healthcare professionals in a leadership role within one of the designated regions, (2) practical experience in the implementation and utilization of the AI application, and (3) proficiency in the Swedish language. The study involved interviews with thirteen healthcare leaders. Healthcare leaders were understood as meaning individuals who oversee as well as coordinate the implementation and use of AI in everyday working situations in healthcare. The emphasis was on the practical aspects of leadership, rather than on the formal titles and roles that have traditionally been associated with leadership. This approach represents an emerging tradition of leadership research [42, 43]. The leaders held a variety of job titles, including business manager, head of unit, development manager/strategist, IT supervisor, and coordinator. Their qualifications were diverse, encompassing physicians, nurses, medical secretaries, and other medical/healthcare professionals, such as physiotherapists or health coaches. A few also had non-medical/healthcare education, that is, they were social workers, had a university degree in healthcare informatics, or had completed tertiary education. All had a role in their organization that entailed managing, coordinating, or overseeing the implementation of the AI application, this type of role had many names i.e., operations managers, development managers/strategists and integration managers.

The healthcare leaders exhibited diverse levels of involvement in the process of implementing the AI for automating medical history-taking and triage. Some were engaged in the implementation across both their own primary care units and other such units, while others focused solely on their own primary care unit or assumed a strategic role rather than an operational one. Approximately half of the sample worked in digital care units, where healthcare services were exclusively delivered via digital means such as video consultations and messaging functions. Conversely, the remaining half were situated in traditional care units, which employed a combination of digital, physical, and telephone appointments, to provide healthcare services. Most leaders operated in primary care units where the AI application had been implemented and in use for over 11 months (see Table 1 in the Method section for a comprehensive overview of participant characteristics).

The recruitment process was facilitated through collaboration with a representative from the company delivering the AI application, who provided contact details for five healthcare leaders meeting the specified criteria. Initial engagement with these leaders occurred via email, resulting in the expression of interest in participating in individual interviews. Employing a snowball sampling technique [49], participants were subsequently requested to identify additional individuals possessing the requisite qualifications and deemed valuable for the study’s objectives. This resulted in an additional eight healthcare leaders consenting to engage in individual interviews, culminating in a final cohort comprising 13 healthcare leaders. Upon consulting with participating senior leaders who possess insights into the pool of eligible interviewees, it was determined that all healthcare leaders actively engaged with the AI application within the three included healthcare regions had been approached to request their participation in the study. Therefore, the data collection was concluded with 13 participants. Comprehensive details of participant characteristics are presented in Table 1.

**Table 1:** Participants’ characteristics (*n* = 13)

**Sex**	
Male	3
Female	10
**Role**	
Operations manager	5
Development manager/strategist	3
Integration managers	5
**Education**	
Physician	2
Nurse	3
Medical Secretary	3
Other medical/healthcare education	3
Non-medical/healthcare education	2
**Type of care unit**	
Digital care unit	7
Traditional care unit	6
**Duration of implementing/using the AI application at the care unit**	
3 years	9
11-18 months	2
3 months	3
**Region**	
Region 1	1
Region 2	6
Region 3	6

### Data collection

The individual interviews were conducted via video communication between November 2022 and March 2023, utilizing a semi-structured interview technique [49]. Two authors, ES, PhD, a postdoctoral researcher, and DT, PhD, a senior lecturer, both equipped with training and experience in qualitative methods, facilitated the process. Importantly, the interviewers had no pre-existing relationship with the interviewees. An interview guide, consisting of open-ended questions, directed the course of the interviews. These questions were structured to explore facets related to the implementation, utilization, and patient safety implications of the AI application. The interview guide underwent a pilot phase with a primary care leader possessing first-hand experience in implementing the AI application prior to the formal adoption. The guide comprised seven themes addressing key aspects of the implementation process, encompassing topics such as (1) the introduction of the AI application, (2) the trajectory of the implementation process, (3) ongoing implementation activities, (4) the impact of the AI application on current work practices, (5) distinctions from previous procedures, (6) effects on daily work for healthcare leaders, and (7) ramifications for healthcare professionals. These themes were tailored to elicit nuanced accounts of leaders’ experiences with the implementation, shedding light on encountered barriers and the strategies employed to surmount them. The interviewers employed prompts, such as ‘Could you elaborate on that?’, in response to the interviewees’ narratives or statements to garner additional information. The interviews ranged from 43 to 67 min with a mean duration of 53 min, all were recorded in audio format and subsequently transcribed for comprehensive analysis.

### Data analysis

The transcribed interviews were analysed with an inductive approach using techniques from thematic analysis [50]. The data was organized using Atlas.ti Web version 9.0 (Scientific Software Development GmbH, Berlin, 2022). Initial codes and themes were developed by one of the authors (ES) and verified for trustworthiness during a feedback session where four of the participants took part. They were asked if they recognized their own experiences in the analysed data, which they confirmed. This type of activity can be considered a form of ‘member-checking’ [59]. To enhance the analysis’ rigor, one author (DT) read the data material and reviewed the analysis including coded extracts, codes, sub-themes, and themes in connection to the entire data material. Afterwards, ES and DT discussed and refined the analysis. The final analysis generated three overarching themes and nine sub-themes. The authors determined that thematic saturation [60] was attained after no new themes were discovered in the data. All authors defined and renamed the sub-themes and themes in the final stage of the analysis.

## Results

### The healthcare leaders’ experiences of barriers to implementing the AI application for medical history-taking and triage and the action they took to navigate around these barriers

A prevailing positive sentiment emerged regarding the application’s dynamic network structure and its efficacy in enhancing patient care in our analysis of the experiences of healthcare leaders in implementing the AI application for medical history-taking and triage. However, we discerned three distinct themes of barriers encountered by the leaders during the implementation process. These barriers manifested in the three domains: healthcare professional-related, organization-related, and technology-related. The first domain included the barriers professional scepticism and resistance, the second involved adapting traditional units to digital care, and the third highlighted inadequacies in AI application functionality and system integration.

**Fig. 2:**
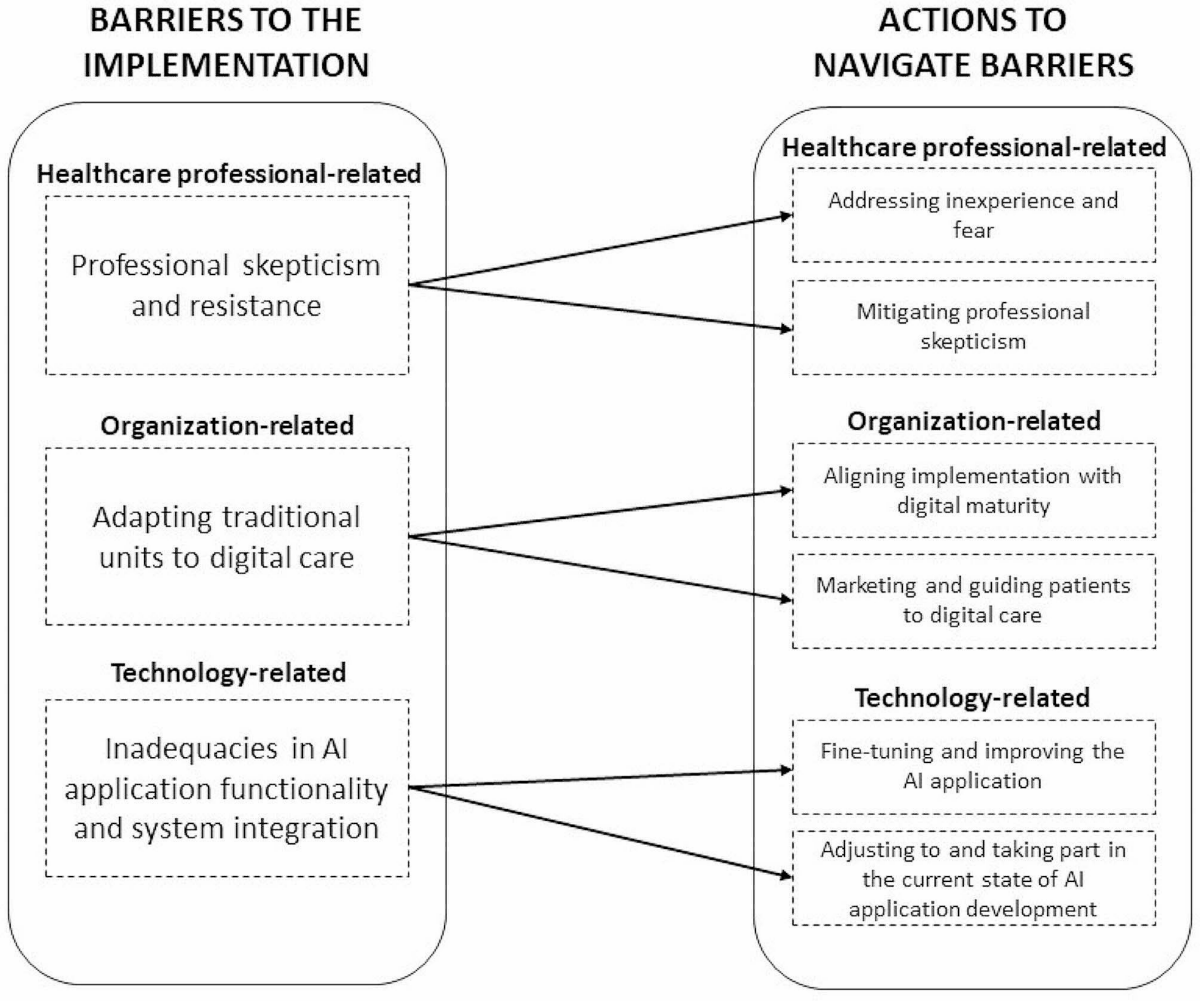
A model of how the healthcare leaders aligned their actions with specific implementation barriers

The healthcare leaders in this study were pioneers in using AI to automate medical history-taking and triage in primary care. They thus had to acquire knowledge through experience and actions over time to overcome barriers when implementing the AI application. The leaders employed actions to address inexperience and fear and mitigate professional scepticism to navigate around barriers in the first domain. They aligned the implementation with digital maturity and engaged in marketing and guiding patients to digital care when addressing barriers within the second domain. The leaders fine-tuned and improved the AI application and adjusted to and took part in the current state of AI application development for the barriers within the third domain. Figure 2 provides a model illustrating how the healthcare leaders strategically aligned their actions with specific implementation barriers. The figure also presents the themes and sub-themes. The ensuing findings delve into the dimensions of these barriers and the actions taken by leaders to overcome them.

### The healthcare professional-related domain

#### Barriers to the implementation: professional scepticism and resistance

The healthcare leaders expressed difficulties in getting primary care professionals to accept and use the AI application when taking the patients’ medical history and assessing proper treatment options for the patients. The resistance towards the AI application among primary care professionals was linked to inexperience, fear, and scepticism, which could also lead to a lack of trust in the application according to the leaders. For example, the healthcare leaders faced resistance from primary care professionals, particularly older nurses unfamiliar with digital care delivery:

*Initially, a few nurses, who are older and who haven’t worked very much with computers and who are not experienced with computers or mobile phones overall but who can handle phone calls and documentation and everything else, found it to be yet another technical system they had to learn and expressed fears of not being able to ask the initial questions during the medical history-taking* (Leader 7)

There were also concerns among these nurses that the AI application might lead to a reduction in their responsibilities and in-person encounters with patients.

The healthcare leaders also highlighted the scepticism pervasive among primary care professionals regarding the necessity and utility of the AI application. Particularly, physicians expressed a belief that the intricacies of taking a patient’s medical history and triaging required elements inherent to the ‘clinical gaze’ and the traditional practices of general medicine:

*I have encountered physicians who, right from the start, said ‘Stop, I don’t read forms, and I don’t read summaries. To take the medical history and see the patient is an art* (Leader 2)

According to the leaders, the physicians perceived these processes as extending beyond mere symptom notation or diagnosis, involving crucial aspects like patient acknowledgement. Their critique of the AI application stemmed from its perceived inability to encompass these nuanced elements, leading to scepticism about its inherent value.

#### Actions to navigate around barriers: addressing inexperience and fear

To address inexperience and fear, the leaders took measures to ensure comprehensive involvement and information dissemination among all primary care professionals prior to and during the AI application launch. Actions to enhance involvement and information dissemination among primary care professionals aimed to offer guidance and support, address concerns and establish trust. These actions encompassed encouraging queries, opinions, and suggestions to enhance application utilization. Specific attention was directed toward particular groups, such as nurses nearing retirement age, providing additional training and support to alleviate apprehensions linked to AI application implementation. The healthcare leaders also arranged different types of network activities for support and exchange of experiences about the implementation and use of the application. This involved meetings where leaders and primary care professionals from various primary care units and the company supporting the AI application training and configuration could meet to exchange experiences and receive support:

*We arrange network meetings where we meet and they are able to share experiences with each other […] for example, there are buttons in the app for certificate and prescription renewal. Users can simply press these buttons, but every user has different ways of how to work with these functions and they share their experiences with each other* (Leader 5)

Additionally, the healthcare leaders in one region had established a ‘super user network’ to offer ongoing guidance and support. This network, comprising proficient users of the AI application, facilitated communication with primary care professionals, ensuring prompt resolution of issues and a channel for reporting technical challenges to higher management and the AI application developers when necessary.

#### Actions to navigate around barriers: mitigating professional scepticism

To mitigate professional scepticism, healthcare leaders emphasized the pivotal role played by primary care professionals in making patient care decisions and were keen on avoiding top-down governance during AI application implementation. These types of actions were deemed essential to foster a sense of ownership and establish trust among employees regarding the application. The AI application was positioned solely as a decision support tool to augment the work of primary care professionals. The healthcare leaders thus showed a willingness to entrust primary care professionals with the autonomy to determine the most effective utilization of the AI application. For example, recognizing that seasoned physicians might choose not to engage with it or that nurses might prefer direct patient calls over messaging or video consultations. Despite the potential efficiency offered by the AI application:

*[…] we tell our nurses: ‘if you think it’s better to call the patient, do it. Pick up the phone and call the patient. You do what you think is best and by which means you should handle things.’ The AI application is a means for the patient to contact us and to give us some information in advance through the medical history-taking function and from that point on it’s us who handle the patient’s case. If that’s by messaging or calling the patient that’s up to the nurse to decide. It has been liberating having that attitude* (Leader 10)

Actions to avoid top-down governance also involved framing the implementation as a ‘grass roots mission’ avoiding forcing the AI tool onto the professionals. Efforts were instead made to encourage voluntary use and to build trust in the application, thereby increasing its use.

### The organization-related domain

#### Barriers: adapting traditional units to digital care

The healthcare leaders highlighted barriers encountered by traditional primary care units in navigating patient cases via web and mobile applications, crucial for utilizing the AI application. These hurdles stemmed from the units’ unfamiliarity with managing the influx of patient cases through the AI application and the difficulty in encouraging patients to embrace its use. Consequently, the way patients interacted with the primary care units’ web and mobile applications became a significant factor influencing the capacity of primary care units to organize their work. While initial expectations envisioned a reduction in phone calls to traditional primary care units post-AI application integration, this was not the reality. In fact, there were instances where patient inquiries increased post-AI integration, generating staffing barriers due to inconsistent patient volumes:

*It’s challenging to manage time dedicated to it. It’s quite inconsistent with the number of people who contact us through the app, making it challenging to plan staffing accordingly* (Leader 12)

Healthcare leaders also noted a significant impact on patients seeking care at traditional primary care units due to their unfamiliarity with using mobile and web applications and automated medical history tools. For example, the AI application’s automated medical history functionality could prove challenging for some patients due to the extensive questionnaire:

*There are pros and cons. One major drawback is that it may make it harder for patients to seek care because there are too many options to go through. This could result in patients not using the application at all or seeking care elsewhere. I think that’s a real disadvantage that we need to take seriously* (Leader 1)

The integration of the AI application thus led to an increase in patient workload across primary care channels. Patients seeking care not only through the AI app but also via other conventional means contributed to the rise in workload.

#### Actions to navigate around barriers: aligning implementation with digital maturity

Successful implementation, as emphasized by healthcare leaders, necessitated aligning implementation with the digital maturity of each primary care unit. These types of actions aimed at tailoring the AI application usage with primary care professionals’ approaches, adapting the technical configuration, and ensuring compatibility with the units’ care delivery. The healthcare leaders underscored that introducing an AI application for medical history-taking and triage should augment existing practices:

*If you say that an AI application will be the first point of contact for patients seeking healthcare, someone unfamiliar with AI might say, ‘That’s not possible. Oh, I don’t want that. I prefer a human to do it’ […] But if you have experience with digital care delivery and know the common reasons patients seek care and how you could utilize this kind of application then you might think: ‘I would really like to have an AI application because it’s safer than a human being. Humans make mistakes all the time, perhaps an AI application won’t make the same mistakes? We should really get one’. You need to be able to see the benefits of when it’s useful* (Leader 6)

The healthcare leaders also highlighted the necessity for primary care units to prioritize enhancing medical history taking and patient triage procedures to effectively integrate the AI application. This approach allowed the units to initiate and lead the implementation process themselves aligning with the leaders’ strategy to frame the implementation of the AI application as a ‘grass roots mission’.

#### Actions to navigate barriers: marketing and guiding patients to digital care

To address patients’ unfamiliarity with using mobile, web and AI applications, healthcare leaders focused on marketing the primary care unit’s web and mobile applications and guiding patients in using the AI application. Marketing efforts were conducted on homepages and, occasionally, through advertising on television. The leaders also emphasized the importance of ensuring positive patient experiences using these platforms, believing it would encourage future utilization:

*We’ve marketed a bit, but mostly depend on natural growth to attract patients to our mobile and web applications […] We think that if we reply to messages quickly, people will spread the word about us. It’s a small community where we live […] They [the patients] might say ‘it’s hard to get through on the phone, but I got great answers when I contacted them this way* (Leader 3)

These types of actions aimed to promote use of the AI application among patients. However, this task was perceived as challenging as the healthcare leaders expressed having little control over how the patients chose to contact them. Another way that the leaders sought to prompt patients’ use of the AI application was to get primary care professionals to address this matter when patients sought care over the phone. This involved primary care professionals educating patients over the phone about the web and mobile applications and guiding and encouraging them to accessing care digitally.

### The technology-related domain

#### Barriers: inadequacies in AI application functionality and system integration

The healthcare leaders reported facing barriers linked to inadequacies in AI application functionality and system integration. This involved challenges when taking patients’ medical history and triaging them due to the AI application’s deficiencies. Additionally, the leaders recognized that the primary care professionals’ utilization of existing digital systems, some of which were obsolete and ineffective, when obtaining medical histories and triaging patients also contributed to the difficulties, not only the AI application.

Healthcare leaders highlighted barriers encountered during patient triage due to deficiencies within the AI application. The AI tool sometimes faced difficulty assessing patient’s conditions accurately, especially when patients did not provide enough information or exaggerated symptoms.

Particularly, the AI application struggled with accurately prioritizing patients with pre-existing conditions, posing a considerable challenge in primary care:

*It’s important to remember that 40% of the population in Sweden has a chronic illness, which poses a challenge […] and then, of course, it becomes difficult to apply the usual symptom flow to someone with an underlying illness and a number of medications* (Leader 2)

As mentioned, technical barriers extended beyond the AI application itself, involving integration with existing digital systems. For example, the primary care’s medical record system did not integrate with the AI application, forcing nurses to manually transfer data, increasing their workload. The healthcare leaders explained that primary care professionals were compelled to use multiple digital systems, some of which were outdated and inefficient. Many of the leaders recognized at the same time the inevitability of the evolution of digital care thus acknowledging the rapid pace of digitization in healthcare and the need for adaptability:

*The government said Sweden must excel in digitization, while our region aims to be the best in Sweden, meaning we need to be the best worldwide. It’s daunting to think that way… but we’re witnessing industry after industry is becoming digitized. So, there’s no going back* (Leader 3)

#### Actions to navigate around barriers: fine-tuning and improving the AI application

To fine-tune and improve the AI application, healthcare leaders engaged primary care professionals, encouraging independent judgment, reporting system shortcomings, and emphasizing that the increased AI application usage would enhance its proficiency. Moreover, some leaders utilized a network of ‘superusers’ maintaining ongoing dialogues with users and the company developing the application. Additionally, the leaders actively contributed to enhancing the AI application’s functionality. For example, they assembled a group of primary care psychologists to improve the triage of patients with mental health conditions initially overlooked by the application. This proactive engagement was perceived as beneficial:

*…some services are just implemented like ‘bang boom’ and then you must accept the situation. Having the opportunity to influence the development [of the AI application] in a direction we want contributes with great knowledge to everyone involved about what happens [with its development] and what the employees at the primary care centres perceive as useful and less useful* (Leader 9)

Some healthcare leaders acknowledged the common belief that AI applications are more advanced than they currently are. They suggested that involvement in their development could ensure that these applications meet the needs of primary care.

Actions to fine-tune and improve the AI application sought to spur improvements to the AI application thereby enhancing its usability and consequently its utilization. However, these types of actions simultaneously fostered increased learning among both leaders and primary care professionals as well as enabled the leaders to gain insight into the perceptions of its usefulness among primary care professionals.

#### Actions to navigate around barriers: adjusting to and taking part in the current state of AI application development

Adjusting to and taking part in the current state of AI application development was another vital action to overcome inadequacies in AI application functionality and system integration. Healthcare leaders argued that integrating up-to-date AI technology into outdated and ineffective digital systems can result in more advantages rather than solely relying on static patient forms and decision-support tools based on rules. The AI application in question could save time during triage procedures, making it advantageous. According to several leaders, it was preferable to utilize the AI application as it was, instead of waiting idly for a perfect system that does not exist in the near future. Furthermore, they pointed to the need to have a long timescale when thinking about the development:

*I believe much will happen in the next few years – something that is very positive – but then, if you’ve been sitting and waiting and thinking that ‘how nice that we didn’t go through all this trouble [with AI applications] before’, then you’ll be disappointed because the tool is a way [of working] but it’s everything else that demands a lot: the trust, the confidence, how we receive it, how we change our way of working. That is the big challenge, I believe – and to be bold enough to go through with it* (Leader 8)

In summary, while healthcare leaders had limited control over the current digital systems, their active involvement in healthcare’s digital and AI advancements aimed to improve current and future operations and contribute to the long-term development of digital systems.

## Discussion

This study investigated healthcare leaders’ first-hand experiences with implementing an AI application for automating medical history taking and triage in Swedish primary care. It provides empirical insights into the barriers to this implementation and how healthcare leaders aligned their actions to overcome them. The barriers and actions manifested in three domains: healthcare professional-related, organization-related, and technology-related, indicating that these are three domains relevant for healthcare leaders when implementing AI in healthcare in general and, more specifically, AI for medical history taking and triage in primary care.

Empirical understanding of healthcare leaders’ experiences in implementing AI in primary care and other healthcare settings is limited [3, 39, 40]. Furthermore, there is a discrepancy in the perspectives of stakeholders in the current research on AI systems. This discrepancy has been identified and a call to address it has been made [61]. Additionally, to the best of our knowledge, empirical research considering healthcare professionals’ perspectives on AI systems for automating medical history-taking and triage is scarce, with only a few examples available [13, 14]. Consequently, this study empirically addresses the discrepancy in stakeholders’ perspectives.

Knowledge about the approaches healthcare leaders take to address barriers tied to AI implementation is scarce [18]. The findings of this study build upon previous theories that emphasize the critical impact of healthcare leaders and their facilitative actions in shaping implementation outcomes [33–35]. Furthermore, the study links leaders’ actions to specific barriers and offers an empirically based understanding of how to deal with them. The findings of this research therefore assume significance by offering empirically-based insights into the nuanced dynamics of overcoming barriers in the implementation of AI in healthcare. The insights also serve as valuable inputs for the development of more broadly applicable strategies for AI implementation in healthcare settings.

The sections below discuss the barriers faced by healthcare leaders when implementing the AI application and the actions they took to navigate around these in terms of the three domains related to healthcare professionals, organisation, and technology. Furthermore, the empirical insights are discussed in terms of how they may inform AI implementation strategies.

### The healthcare professional-related domain

With regard to healthcare professional-related barriers in implementing the AI application automating for medical history-taking and triage, our empirical findings substantiate the pervasive anxieties and scepticism among primary care professionals, serving as significant barriers to the integration of AI in healthcare, which is consistent with previous research [13, 21, 22, 62]. The observed reluctance, as articulated by the healthcare leaders in our study, was intricately tied to primary care professionals’ inherent lack of trust in AI’s performance, coupled with a steadfast belief in their own expertise – an observation that echoes the findings of prior research emanating from the perspectives of both healthcare professionals [13] and leaders [39].

The healthcare leaders undertook a comprehensive array of techniques in addressing implementation barriers stemming from primary care professionals’ scepticism, fear, and limited experience. These included active involvement, peer-to-peer learning initiatives, recourse to supplementary resources such as the AI application vendor and provided primary care professionals with increased flexibility in using the AI application. Additionally, some of the healthcare leaders in this study, adopted innovative methods such as superusers. Similar innovative methods have been proposed by previous research as a means of facilitating the implementation of digital triage applications in primary care [22, 63].

The findings indicate that AI implementation strategies in this healthcare professional-related domain should address healthcare professionals’ fear and scepticism towards AI’s role in interpreting data and contributing to the creation and evaluation of new knowledge claims [15, 16] as well as supporting professionals in their core medical responsibilities [17, 18]. These characteristics distinguish AI from other commonly studied technologies. Our findings indicate that adequate training and ability for users to comprehend the AI application [64–66] as well as novel approaches, such as superusers [22], could be beneficial in overcoming such implementation barriers, which is consistent with prior research.

### The organization-related domain

The healthcare leaders in our study indicated organization-related barriers related to the traditional primary care units’ ability to manage the incoming flow of patients via web and mobile applications, which was crucial for utilizing the AI application. In general, AI is believed to have the potential to reduce workload of healthcare professionals working with medical history-taking triage and contribute to safer and more efficient triage [10–14]. On the other hand, the healthcare leaders in our study expressed that the implementation of the specific AI application for medical history taking and triage sometimes increased the workload for healthcare professionals. The increase in workload was attributable to patients seeking care through both the AI application and other conventional means, as well as the lack of integration between the AI application and other digital systems. The findings suggest that in order for AI to reduce the workload of healthcare professionals in medical history-taking and triage, its implementation must be tailored to the digital capabilities of each unique primary care setting. This is consistent with previous research on other digital platforms for managing patient flow [67, 68]. Another barrier faced by the leaders was the deficiency in both the familiarity and proficiency of their patient populations in the utilization of AI technology. This barrier is congruent with previously identified patient-related barriers for implementing AI for medical history-taking and triage, including limited comprehensiveness within certain patient groups [14], limited patient eHealth literacy [14, 22] and the confluence of a modest user base [22].

The healthcare leaders proactively addressed barriers associated with the primary care units’ unpreparedness for innovation and change by tailoring actions to the diverse levels of maturity and motivation evident among the primary care units. Concurrently, the leaders fostered an environment wherein primary care professionals actively advocated the advantages of the AI application to patients through marketing strategies deployed via web and phone applications. These actions emerged as essential facets in overcoming implementation barriers when coupled with primary care professionals assuming an educational role in instructing patients on optimal application use.

These findings suggest that AI implementation strategies in the organizational domain should address barriers linked to healthcare organizations’ lack of digital maturity, including patients’ unfamiliarity with digital care. The implementation of AI is conditioned by an organizations’ willingness and ability to implement it [39]. According to our findings, successful implementation of AI for medical history taking and triage in primary care requires two innovation-specific capacities [69]: the healthcare setting’s digital maturity and the patients’ familiarity with digital care. To address barriers related to these capacities, our findings, in line with previous research, suggest that acknowledging the organizational context and effectively communicating AI’s benefits to end-users [64, 65] are two ways to promote successful implementation.

### The technology-related domain

The healthcare leaders highlighted technical barriers in terms of the AI application and its inadequate integration with existing technical systems when it came to automating medical history-taking and triaging patients, which is consistent with previous research [13, 14, 21, 22]. Our study showed in particular that technical barriers often impede the ability of AI applications to triage patients with underlying diseases or highly diverse backgrounds, a challenge that has also been reported in other clinical settings [13]. The potential for AI systems to automate medical history taking and triage in first-line care is high, given that the field faces global challenges in meeting the needs of patients [70, 71]. However, AI applications in these care settings may face difficulties due to the diverse patient demographics they serve, as highlighted by our research and previous studies [70, 71].

To overcome the inadequacies of the AI application’s patient triaging capabilities and the subpar integration of the system, coupled with the rapid advancements in digital care and AI technology in healthcare, the healthcare leaders took a proactive approach in enhancing the AI application and staying abreast of the latest developments in digitalization and integration of AI in healthcare. This involved remaining up to date with current advancements and anticipating long-term AI advancements. However, the healthcare leaders did not mention working on the integration of the AI application into current information systems, which is somewhat surprising as integration with existing technical systems has been put forward as a significant aspect for successful implementation of this type of AI application [14].

The findings suggest that implementation strategies in this technology domain should address shortcomings of the AI applications as well as of their integration with existing technical systems. Nevertheless, the findings also highlight that there are aspects of the implementation that healthcare leaders have little control over. The implementation of AI has been considered to be more complex than that of other digital technologies due to its unpredictability, the lack of empirical evidence, and the difficulty in conceptualizing it [23]. This challenge is further compounded by the rapid evolution of AI applications within the healthcare domain, as was expressed by the healthcare leaders in this study. Although our findings do not assess the effectiveness of the healthcare leaders’ actions, their approach of adapting the implementation to local-specific barriers linked to not only technical barriers, but also barriers related to healthcare professionals and the organization, suggests that the rapidly evolving nature of AI applications in healthcare may require implementation strategies that allow for a certain degree of improvisation. Such improvisation involves the ability to navigate and respond to the demands of the dynamic local landscape based on practical wisdom and tacit understanding [72].

### Strengths and limitations

The study only involved 13 healthcare leaders. However, the snowball sampling technique [49] employed in this study did not yield more participants. Furthermore, the senior leaders who possessed insights into the pool of eligible interviewees indicated that all healthcare leaders actively engaged with the AI application within the included regions had been approached. Therefore, it can be asserted that all eligible participants were approached for participation in the study. This signifies a methodological thoroughness reflective of the study’s intrinsic purpose. Furthermore, it is imperative, however, to underscore that in qualitative research, the significance of sample size transcends mere numerical augmentation, pivoting instead the importance of a sample appropriate for the study objectives [73]. Furthermore, the credibility of the study is strengthened by all participants having meaningful experience of the implementation of the AI system for automating medical history-taking and triage [74]. While the study’s reliance on experiences with a single AI application from one supplier might appear limiting, exploring multiple applications for automated medical history-taking and triage would introduce significant technical variations. This could risk data discrepancies stemming from diverse user experiences tied to these technological differences. Hence, the study’s focus on a unique AI application, specifically the only one in Sweden employing a Bayesian network for triage rather than a fixed decision tree, constitutes a strength. This approach highlights the potential and unique attributes associated with the introduction of AI in this context. To strengthen the analysis of the participants’ account we conducted a form of ‘member-checking’ [59]. The participants confirmed the alignment of their experiences with the interpreted data during a feedback session, reinforcing the accuracy of the analysis. This form of validation adds credibility to the findings by ensuring the authenticity of the derived themes. Additionally, to strengthen the analysis’ rigor, an iterative process involving multiple authors was employed. However, while member-checking reinforced the credibility of interpretations, it is important to note that the sample size for this validation technique was limited to a subset of participants, which could potentially influence the trustworthiness of the findings. Finally, during the period of data collection for this study, the AI application was either being implemented or used in four Swedish regions. Three of these regions were included, while a fourth region was not included due to time and resource limitations. It should be noted that had the fourth region been included in the study, it would possibly have strengthened the findings in terms of providing corroborating support and more regional variation.

## Conclusion

In conclusion, this study focused on healthcare leaders’ first-hand experiences with implementing an AI application for medical history-taking and triage in Swedish primary care. The findings provide empirical insights into the barriers to this implementation. It highlights how healthcare leaders aligned their actions to overcome these barriers across three domains: healthcare professional-related, organization-related, and technology-related. The findings underscore the importance of these domains relevant for healthcare leaders engaging in AI implementation efforts. While progress was evident in overcoming healthcare professional-related and organizational-related barriers, unresolved technical complexities emphasize the need for AI implementation strategies to consider how healthcare leaders handle barriers in situ based on practical wisdom and tacit understanding. This underscores the multifaceted nature of implementation barriers and the essentiality of a holistic approach for successful integration of AI in healthcare practices. The findings illustrate the importance of empirical research on diverse AI applications and various healthcare settings for their implementation, steering towards more informed, robust, and generally applicable strategies to guide the implementation of AI for medical history-taking and triage in primary care.

### Electronic supplementary material

Below is the link to the electronic supplementary material.

Supplementary Material 1

## Data Availability

The interviews analysed in this article are not publicly available because the study participants did not give consent for their data to be shared publicly. However, anonymized interview transcripts are available from the corresponding author upon reasonable request.
